# Nomogram Analysis and Internal Validation to Predict the Risk of Cystobiliary Communication in Patients Undergoing Hydatid Liver Cyst Surgery

**DOI:** 10.1007/s00268-020-05661-5

**Published:** 2020-07-09

**Authors:** Zhan Wang, Jin Xu, MingQuan Pang, Bin Guo, XiaoLei Xu, HaiJiu Wang, Ying Zhou, Li Ren, LingQiang Zhang, Jie Ma, HaiNing Fan

**Affiliations:** 1 Department of Hepatopancreatobiliary Surgery, The Affiliated Hospital of Qinghai University, Xining, China; 2 Qinghai University, Xining, China; 3 Department of Otorhinolaryngology, The Affiliated Hospital of Qinghai University, Xining, China; 4 Qinghai Province Key Laboratory of Hydatid Disease Research, 29 Tongren Road, Xining, 810001 Qinghai China

## Abstract

**Purpose:**

Biliary leakage caused by cystobiliary communication (CBC) is a common clinical concern. This study sought to identify predictors of CBC in hepatic cystic echinococcosis (HCE) patients undergoing hydatid liver cyst surgery and establish nomograms to predict CBC.

**Methods:**

A predictive model was established in a training cohort of 310 HCE patients diagnosed between January 2013 and May 2017. Upon revision of the records of clinical parameters and imaging features of these patients, the lasso regression model was used to optimize feature selection for the CBC risk model. Combined with feature selection, a CBC nomogram was developed with multivariable logistic regression. C-index and calibration plots were used to analyze and evaluate the discrimination and calibration. The net benefit and predictive accuracy of the nomogram were performed via decision curve analysis (DCA) and receiver operating characteristic (ROC) curve. An independent validation cohort of 132 patients recruited from June 2017 to May 2019 was used to evaluate the practicability of the nomogram.

**Results:**

Predictors contained four features, namely alkaline phosphatase (ALP), glutamyl transpeptidase (GGT), cyst size and cyst location. The C-index of the nomogram is 0.791 (95% CI, 0.736–0.845), while the C-index verified by bootstrap is 0.746, indicating high prediction accuracy. The area under the curve (AUC) of the CBC in training was 0.766. ROC curve analysis demonstrated high sensitivity and specificity. Decision curve analysis confirmed the CBC nomogram was clinically useful when the intervention was determined at the non-adherence possibility threshold of 8%.

**Conclusion:**

The nomogram developed using the ALP, GGT, cyst size and cyst location could be used to facilitate the CBC risk prediction in HCE patients.

## Introduction

Hepatic cystic echinococcosis (HCE), caused by *Echinococcus granulosus*, is a zoonotic parasitic disease. The epidemic area of HCE covers the entire planet with the exception of Antarctica [1]. The occurrence of cystic echinococcosis (CE) can be caused by human consumption of food contaminated by insect eggs. Most infections affect the liver (about 75–80%), a few affect the lung (about 8–15%) and the rest are distributed in the kidney, spleen, omentum, pelvic cavity, brain, etc. [2]. Surgical treatment is commonly used for CE, but regardless of the type of operation used, bile leakage is the most common complication after CE surgery [3]. In fact, it is one of the main causes of increased mortality and prolonged hospital stay in patients with cystic echinococcosis.

The main cause of bile leakage in patients is the existence of cystobiliary communication (CBC), which can be divided into two types: frank fistula and occult fistula [4]. The incidence of frank fistula is about 3–7% [5]. Although frank fistula can be diagnosed by B-mode ultrasound and CT scan before surgery, misdiagnosis is a common problem, resulting from the heterogeneity of training and expertise of the medical staff, as well as the disparity of medical expertise between remote areas and provincial capital cities. In turn, the incidence of occult fistula is about 10–37% [6], and it usually develops without any clinical symptoms, may be found during surgery or may be diagnosed after postoperative bile leakage, thereby favoring the occurrence of abdominal infection. The occurrence of bile leakage seriously affects the postoperative recovery of patients with CE, and it is an important cause of postoperative complications and prolonged hospitalization. The treatment of postoperative bile leakage has increased the financial burden of patients and occupied precious medical resources which are scarce in areas of high disease incidence.

Therefore, early prediction of the occurrence of CBC in patients with CE is of great significance, and there are few reports on prediction models of CBC of cystic hydatid. This study mainly explores the independent risk factors affecting the occurrence of CBC in hepatic cystic echinococcosis and establishes a nomogram model for individualized CBC risk prediction in CE patients, so as to help clinicians in high-risk areas of echinococcosis find high-risk patients of CBC early and reduce the occurrence of postoperative complications.

## Methods

### Study design and patient population

The study was approved by the Medical Ethics Committee of the Affiliated Medical Hospital of Qinghai University. All clinical data are strictly confidential. Patients were not required to agree with review of their medical records, as this is an observational retrospective study and does not infringe the rights of patients. Patients with HCE hospitalized for the first time in the Affiliated Hospital of Qinghai University from January 2013 to May 2019 were enrolled as the primary study subjects.

Clinical data of 342 CE patients treated surgically were collected in the Affiliated Hospital of Qinghai University from January 2013 to May 2017. According to the inclusion and exclusion criteria, 310 patients were included as the training cohort. Data from 171 patients were collected from June 2017 to May 2019, and a total of 132 patients were included as the independent verification cohort under the same conditions. This study strictly follows the STROBE observational study list. Before the study, two hepatobiliary surgeons with more than five years of experience were trained in data collection protocols, to guarantee that data from the same hydatid patient were collected by the same doctor. The data were then entered by double check to ensure the accuracy of the recorded data.

### Data collection

In this study, the clinical characteristics that came from laboratory examinations, imaging examinations and surgical records were: (i) age, sex, height and weight; (ii) alanine aminotransferase (ALT), alkaline phosphatase (ALP) and glutamyl transpeptidase (GGT); (iii) diameter of hydatid cyst and location of cyst (portal area (I, III, IVb and V) and non-portal area (II, IVa, VI, VII and VIII)); and (iv) patients with biliary leakage in the surgical records. In the absence of biliary leakage during operation, the course of the record of biliary leakage was checked.

*Inclusion criteria of patients* (i) CE diagnosed by preoperative CT or MRI and pathological diagnosis after operation (according to expert consensus on the diagnosis and treatment of hepatic cystic and alveolar echinococcosis, 2015 edition); (ii) treated by total cystectomy, partial pericystectomy; and (iii) without other tumors and without preoperative antineoplastic therapy.

*Exclusion criteria of patients* (i) lack of definite imaging and pathological diagnosis or unclear diagnosis; (ii) distant metastasis; (iii) complicated alveolar echinococcosis; (iv) severe cardiovascular and/or cerebrovascular diseases or severe hepatorenal insufficiency; and (v) hepatitis B and hepatic dysfunction during active phase or obstructive jaundice caused by non-echinococcal factors.

### Statistical analysis

Statistical analysis was performed by R software (version 3.5.3) and SPSS 24.0 (IBM, USA). Continuous variables were expressed as mean ± SD, and categorized values were expressed by frequencies or percentage. Student’s *t*-test was used to analyze differences of variance. All statistical tests were bilateral. Categorical data as frequencies were analyzed by Chi-squared test. *P* < 0.05 was considered significant. The least absolute shrinkage and selection operator (LASSO) [7] method was used to select the best predictive characteristics of risk factors from CE patients with CBC. Nonzero features were selected in the LASSO regression model. Then, the selected feature was used to establish the prediction model by the multivariate logistic regression analysis. Internal validation is performed using independent validation datasets.

All potential predictors are used to establish CBC risk prediction models through retrospective studies. A calibration curve was drawn to evaluate the calibration of CBC nomogram. C-index was used to evaluate the discrimination of the model. CBC nomogram was validated by boot validation (1000 boot resampling) to calculate the relative corrected C-index. By quantifying the net benefits under different threshold probabilities in the CBC cohort, the decision curve was analyzed to determine the clinical effectiveness of CBC nomogram. The calibration of the CBC nomogram was assessed by calibration curves. The receiver operating characteristics (ROC) curve was used to evaluate the diagnostic value of the model for discriminating CBC from non-CBC to determine the cutoff value for assessing accuracy, sensitivity and specificity. To quantify the net benefits, the clinical usefulness of the CBC nomogram was conducted by decision curve analysis.

## Results

### Clinical characteristics of the patients

After excluding patients who did not meet the inclusion criteria, 310 patients with CBC in the training cohort and 132 patients in the validation cohort were eventually included. The baseline characteristics of the two cohorts are listed in Table 1. In the training cohort, 87 patients had CBC (28.1%). There were significant differences in ALP, GGT, cyst diameter and cyst location between the two groups (*P* < 0.05). There was no significant difference in age, sex, height, weight, ALT, TBIL, ALB, HGB, PLT, EOS and number of cysts between the two groups (*P* > 0.05). In the verification cohort, 34 patients had CBC (25.8%). The comparison between the two groups in the validation cohort was consistent with the training cohort (Table 1).

**Table 1:** Characteristics of patients included in this study

Variable	Training cohort (*n* = 310)		*P* value	Validation cohort (*n* = 132)		*P* value
	Non-CBC (*n* = 223)	CBC (*n* = 87)		Non-CBC (*n* = 98)	CBC (*n* = 34)	
Clinical parameters						
Age	44.47 ± 14.01	46.13 ± 14.38	0.353	48.71 ± 15.08	51.44 ± 13.78	0.720
Gender						
Male	92	49–9	0.095	32	13	0.167
Female	131	36 + 9		71	16	
Height	165.45 ± 7.39	165.94 ± 7.64	0.376	163.81 ± 7.88	164.85 ± 7.42	0.302
Weight	59.63 ± 11.17	62.70 ± 10.26	0.112	62.70 ± 10.26	59.97 ± 9.44	0.964
Laboratory parameters						
ALT (U/L)	47.44 ± 32.81	70.70 ± 68.58	0.066	44.77 ± 33.91	59.85 ± 57.58	0.785
AST (U/L)	50.97 ± 44.27	62.10 ± 58.79	0.068	51.66 ± 41.08	68.76 ± 51.17	0.086
ALP (U/L)	125.02 ± 90.81	216.75 ± 149.87	<0.001	129.34 ± 78.75	134.56 ± 117.60	0.013
Normal	190	36	<0.001	81	14	0.01
Up to 2 times	39	23		16	10	
Between 2 and 3 times	5	13		2	6	
Between 3 and 5 times	3	11		3	3	
5 times and above	2	4		0	1	
GGT (U/L)	66.70 ± 71.22	114.44 ± 130.39	0.015	66.15 ± 63.21	91.59 ± 89.45	0.02
Normal	150	32	<0.001	63	10	0.035
Up to 2 times	41	19		27	10	
Between 2 and 3 times	13	16		8	4	
Between 3 and 5 times	11	9		2	2	
5 times and above	8	11		2	3	
TBIL (μmol/L)	11.50 ± 16.68	22.83 ± 40.76	0.097	13.86 ± 18.50	24.70 ± 47.22	0.205
ALB (g/L)	36.21 ± 6.12	36.24 ± 5.75	0.860	34.54 ± 4.29	33.97 ± 5.77	0.688
HGB (g/L)	132.43 ± 20.02	129.92 ± 21.32	0.332	140.17 ± 9.62	138.71 ± 8.90	0.571
PLT (10^9/L)	218.86 ± 58.98	223.55 ± 57.51	0.526	248.2 ± 49.59	239.68 ± 58.38	0.390
EOS (10^9/L)	0.41 ± 0.30	0.38 ± 0.31	0.480	0.37 ± 0.28	0.53 ± 0.37	0.174
Imaging parameters						
Cyst size	9.03 ± 2.34	9.97 ± 6.70	0.003	9.39 ± 2.52	9.80 ± 1.86	0.01
<7.5 cm	49	17	0.729	18	6	0.665
7.5–8.4 cm	37	9		16	3	
8.5–9.4 cm	45	10		15	4	
9.5–10.4 cm	32	13		20	6	
10.4–11.5 cm	22	6		12	5	
>11.5 cm	47	18		17	10	
Location of cyst						
Hilar	80	53	<0.001	38	21	0.001
Peripheral	143	34		65	8	
Number of cysts						
Single	149	56	0.682	67	21	0.525
Multiple	74	31		36	13	

*ALP* alkaline phosphatase, *GGT* glutamyl transpeptidase, *ALT* alanine aminotransferase, *AST* aspartate aminotransferase, *TBIL* total bilirubin, *ALB* albumin, *HGB* hemoglobin, *PLT* blood platelet, *EOS* eosinophil

### Feature selection and model development

**Fig. 1:**
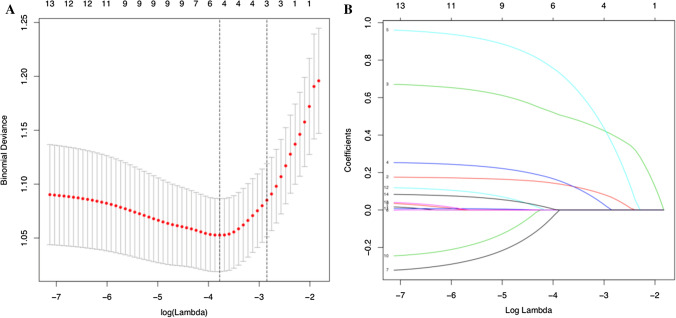
Clinical feature selection by the LASSO. **a** Optimal parameter (lambda) selection by LASSO used fivefold cross-validation via minimum criteria. The minimum criteria and the one SE of the minimum criteria (the 1-SE criteria) are used to draw the dotted vertical line at the optimum value. **b** LASSO coefficient profiles of the 17 variables plotted against the log(lambda) sequence. Drawing vertical lines by optimum lambda values of four nonzero coefficients through fivefold cross-validation

**Fig. 2:**
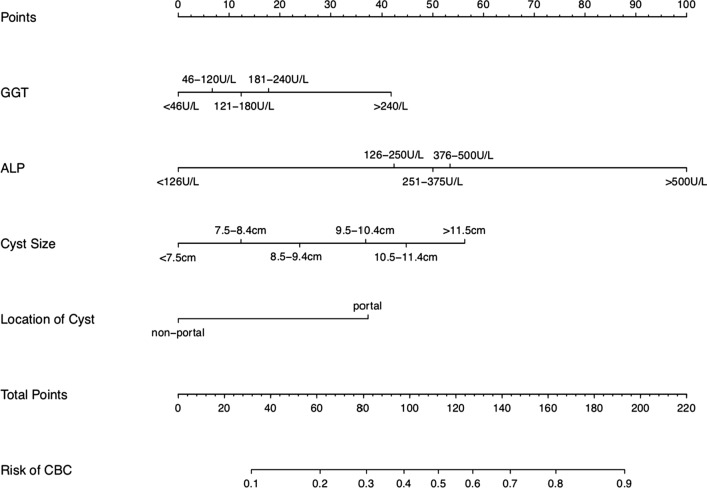
A nomogram to predict the cystobiliary communication (CBC) of hepatic cystic echinococcosis patients. The nomogram was developed in the cohort, using GGT, ALP, cyst size and cyst location. Abbreviations: GGT, glutamyl transpeptidase; ALP, alkaline phosphatase

Seventeen high-dimensional clinical data included in LASSO regression were reduced to three potential predictors on the basis of 310 patients in the training cohort. These features included ALP, GGT, cyst diameter and cyst location (Fig. 1). The results of the multivariate logistic regression analysis are given in Table 2. According to the above independent predictors, the developed nomogram is presented in Fig. 2.

**Table 2:** Multivariate logistic regression analysis of prediction factors for CBC in CE

Variable	*β*	OR (odds ratio)	95% CI	*P* value
ALP	0.585	1.796	1.276 ~ 2.527	0.001
GGT	0.183	1.201	0.916 ~ 1.575	0.184
Location of cyst	0.977	2.657	1.530 ~ 4.616	0.001
Cyst size	0.264	1.302	1.029 ~ 1.647	0.028

*ALP* alkaline phosphatase, *GGT* glutamyl transpeptidase, *CI* confidence interval

### Predictive ability and performances of CBC risk nomogram

**Fig. 3:**
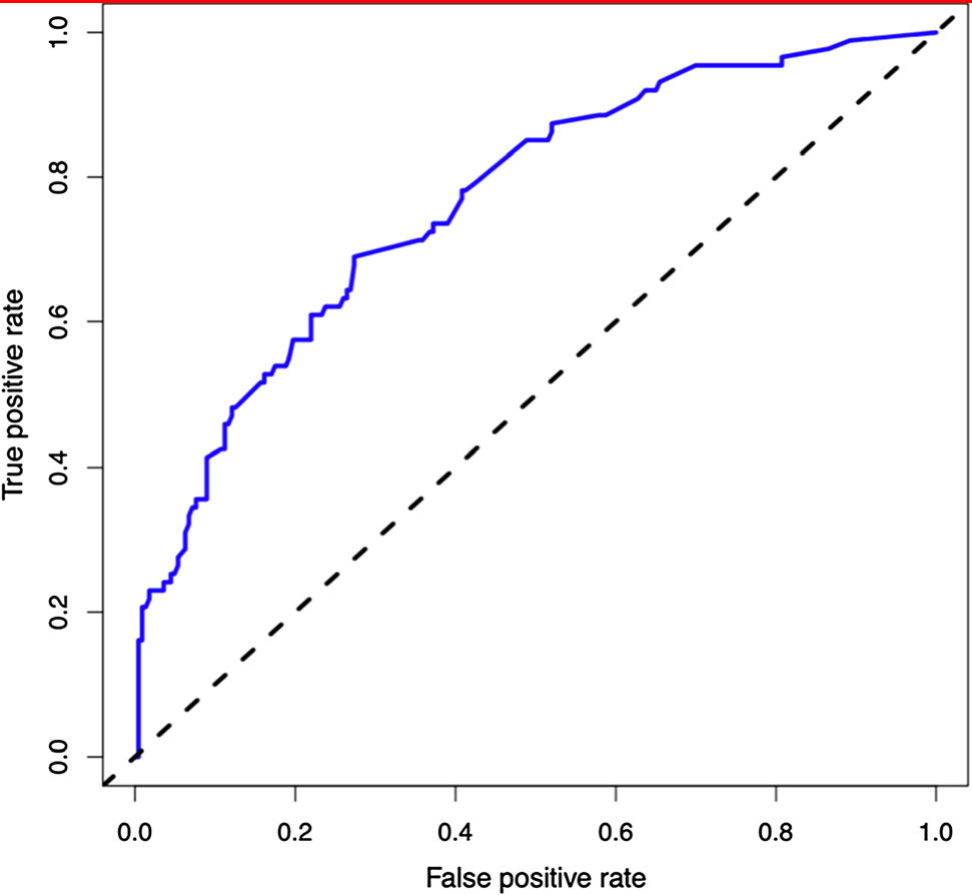
ROC analysis of the nomogram for cystobiliary communication. ROC curve for discrimination in the training cohort. The AUC of the nomogram was 0.766, demonstrating very good prediction performance. Abbreviations: ROC, receiver operating characteristic; AUC, area under curve

**Fig. 4:**
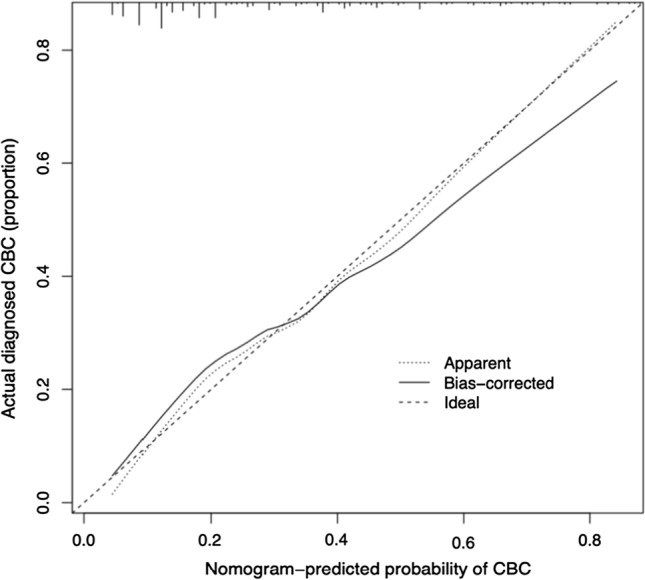
Calibration curves of the cystobiliary communication (CBC) prediction nomogram. The predicted cystobiliary communication risk is shown on the *X*-axis. The actual diagnosed cystobiliary communication is shown on the *Y*-axis

The C-index was calculated to evaluate the predictive ability of the model, and the resulting value was 0.791 (95% CI, 0.736–0.845). Bootstrap was used to verify the over-fitting of the estimation model. The C-index was 0.746 after calibration, which showed that the nomogram had good prediction accuracy. The prediction performance and ROC curves of the CBC risk nomogram are shown in Fig. 3. The model demonstrated valuable prediction performance with AUC of 0.766, which suggested the model has good discrimination capabilities. Apparent performance reflects good predictive ability in the CBC risk nomogram. The calibration curve of the model is in good agreement with the observed results in Fig. 4.

### Presentation of a nomogram and clinical use

**Fig. 5:**
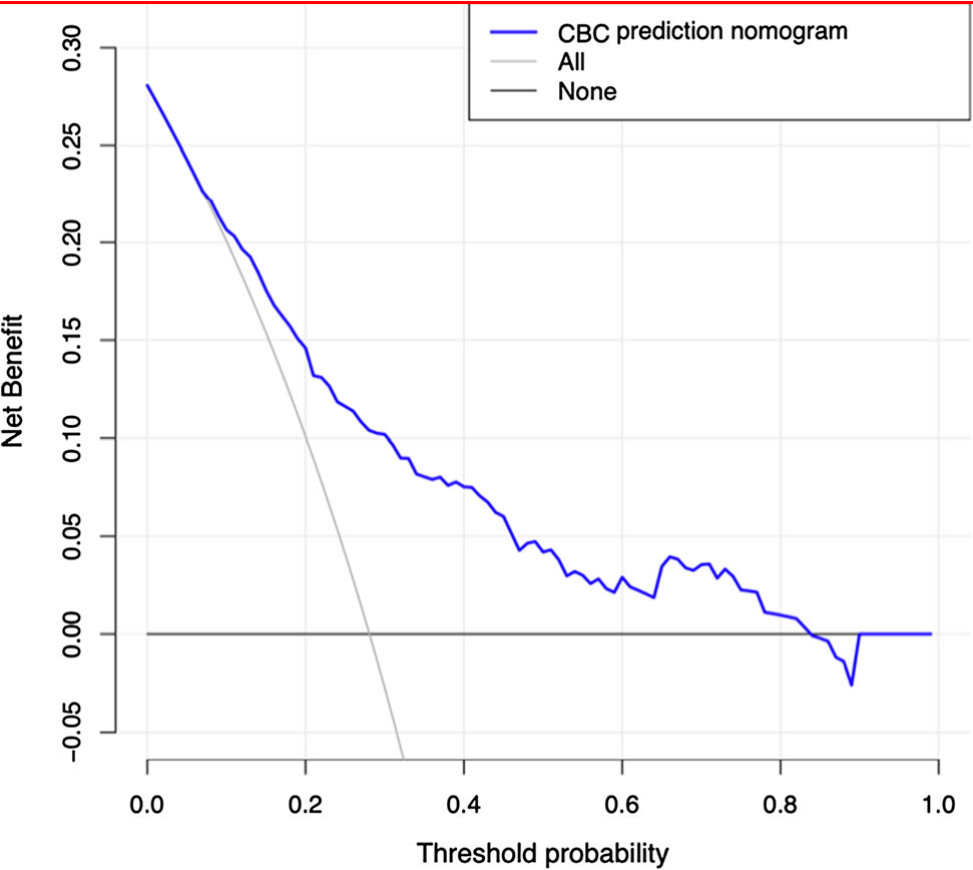
Decision curve analysis (DCA) for the cystobiliary communication (CBC) nomogram. The blue line represents the cystobiliary communication nomogram. The thin solid line represents the hypothesis that all patients had cystobiliary communication (CBC). The thick solid line represents the hypothesis that no patients had CBC. The DCA in the validation cohort demonstrated that if the threshold probability is between 0.08 and 0.83, the use of the nomogram to predict CBC is more beneficial than treating all or no patients

The decision curve analysis for the CBC nomogram is presented in Fig. 5. The decision curve reported that the threshold probability of the net benefit superior to the baseline ranged from 8–83%. Using this CBC nomogram to predict the risk of CBC adds more benefit than portrayed by Fig. 5. On the basis of weighing the net benefit of differentiating threshold probability, a nomogram can easily make timely personalized pretreatment clinical decisions for different risk groups.

## Discussion

CBC is common in hepatic hydatid cysts. Postoperative bile leakage caused by CBC is the most important cause of morbidity and mortality in HCE patients. There are two theories about the pathogenesis of CBC. First, hydatid cysts gradually compress the bile duct wall, leading to necrosis and eventually CBC. Second, the free radicals of small bile ducts in the cystic wall produce high intracystic pressure, which leads to atrophy and subsequent bile duct free radical rupture. In previous studies such as Kayaalp et al. [8] and Demircan et al. [9], the prevalence of CBC was 27% and 28.4%, respectively. In this study, the incidence of CBC was 28.1%. Although doctors carefully search for and sew the internal fistula during CE surgery, postsurgery bile leakage is still hard to avoid, which is mostly due to the existence of occult CBC. CBC is difficult to diagnose by routine preoperative examination, mainly because its preoperative symptoms and examination results are not obvious. Another reason for this is that the pressure of the biliary system is lower than that of the hydatid cyst. The pressure of a normal biliary tract system is 15–20 cm H_2_O, and there is about 35 cm H_2_O pressure in the cystic cavity of hydatid cysts, which is one of the indicators of cystic hydatid survival [4]. Therefore, although occult CBC exists, bile will not flow into the cyst. However, after hydatid surgery, external pressure disappears, necrotic tissue falls off, and bile will enter the abdominal cavity directly through the fistula, leading to secondary abdominal infection.

As a statistical model, the nomogram [10] has the advantages of accuracy, repeatability, visualization and no need for computer software intervention. It enables clinicians to make standardized clinical decisions, and thus, it is becoming one of the most commonly used assistant tools in clinical practice. This study is the first to apply the nomogram to CBC. In this current study, we developed and validated a nomogram as a new method for diagnosing CBC. The chart contains four items: ALP, GGT, cyst diameter and hilar cyst location. Internal validation in the cohort demonstrated good discrimination and calibration power; in particular, our high C-index and AUC identified that the nomogram can be widely and accurately used. A total of 17 candidate variables were used to construct the nomogram, and this number was reduced to four potential predictors by using LASSO regression. LASSO is suitable for analyzing a large number of clinical factors and avoiding over-fitting. Our nomogram indicates that the presence of ALP and cyst diameter may be a good predictor of CBC. In addition, the nomogram can be used as a useful tool for identifying patients at high risk of CBC. Therefore, it will increase the possibility of early intervention for high-risk patients, especially in primary hospitals in areas with uneven distribution of medical resources and echinococcosis high-incidence areas.

El Malki et al. [11] predicted CBC by classification and regression tree based on the morphology, jaundice, age and history of echinococcosis examined by B-mode ultrasonography. Although the specificity of the model was 93.3%, the sensitivity of the model was 39.6%, and there was no evaluation of blood samples. The prediction model was not intuitive, and its clinical application was cumbersome and could not be widely used. At present, CBC-related prediction studies are mostly focused on the analysis of risk factors. Sex, ALP, GGT, ALT, cyst diameter, TBIL, eosinophils and number of cysts can all be used as predictors of CBC, but the risk factors for multiple reports are not consistent. Demircan et al. [9] pointed out that high ALP levels in patients are a strong risk factor for cystic echinococcal bile leakage. Surmelioglu et al. [12] divided ALP into five grades (normal, 2, 3, 4, 5 and >5) in clinical studies of complications associated with cystic echinococcosis biliary leakage. The results suggest that the risk of CBC increases by 2.5 times for every grade of ALP. In current results, ALP was also divided into five grades. The results showed that the risk of CBC increased by 1.796 times for every grade of ALP. This was consistent with the results of other scholars and confirmed that ALP was a risk factor for CBC in cystic echinococcosis.

The size of hydatid cysts has been considered as an important predictor of biliary leakage. Atli et al. [13] pointed out that a diameter of hydatid cyst >14.5 cm was an important predictor of biliary leakage. Demircan et al. [9] suggested that cyst diameter >8.5 cm was a predictor of bile leakage. Kilic et al. [14] divided the cyst diameter into six groups. When the cyst diameter was 7.5 cm, the specificity and sensitivity of bile leakage were 73% and 79%, respectively. Therefore, it was pointed out that a cyst diameter >7.5 cm was a risk factor for bile leakage. In this study, the cyst diameter was divided into four grades (<7.5, 7.5–8.4, 8.5–9.4, 9.5–10.4, 10.5–11.4 and ≥ 11.5). The results showed that the risk of bile leakage increased 2.503 times for each grade of cyst diameter. Dziri et al. [15] divided the liver segments into two strata (II, VII, VIII and III, IV, V, VI) according to the position of the cyst relative to the diaphragm. They did not divide the upper (IVA) and lower (IVB) parts of the IV segment. The results showed that there was no difference between the two groups in the occurrence of bile leakage. In contrast, Perdomo et al. [16] reported that different cyst locations were most likely to cause bile leakage. The closer the cyst was to the portal, the more serious the complications were. Cuneyt et al. [17] divided hepatic segments into (I, III, IVb, V, VI) hilar cysts and (II, IVa, VII, VIII) non-hilar cysts. It was found that the location between hydatid cysts and hilar cysts was a risk factor for biliary leakage and postoperative complications. This may be due to the natural distribution of intrahepatic bile ducts. The distribution of intrahepatic bile ducts is relatively dense in the hilar part of the liver, from the middle lobe of the liver to the left and right lobes of the liver.

These results are consistent with the results of current data. According to Cuneyt's method, the cyst locations were divided into hepatic hilar cysts and non-hepatic hilar cysts. The risk of bile leakage was 2.657 (95% CI: 1.530–4.616) when the cysts were located in the hilar region. It was concluded that the presence of hydatid cysts in the hilar region could be a risk factor for bile leakage. Kayaalp et al. [8] pointed out that 31% of CE patients with CBC had elevated GGT levels. The results of Langer [18] and Safarioleas [19] were consistent with the results of Kayaalp's study. In these studies, the ratio of patients with elevated GGT was 30% and 34%, respectively. Although GGT had no statistical significance in the multivariate analysis in this current study, GGT is still used as a potential predictor by LASSO regression. Based on the conclusions of the above scholars and the results of LASSO regression, in this study GGT was included in the nomogram for CBC prediction.

Nakeeb et al. [5] found that although ALT levels in the cystic echinococcal bile leakage group increased significantly before operation, multivariate analysis showed that ALT was not significant as a predictor of bile leakage, which was consistent with the results of current data. Thus, LASSO regression did not include ALT as a predictor. Demircan et al. [9] pointed out that total bilirubin levels higher than 17.1 μmol/L and eosinophil (EOS) levels greater than 0.9 could be used as an independent predictor of CBC occurrence. However, Atahan et al. [20] found that EOS could not be used as an independent predictor of CBC occurrence, which is consistent with this study. Bircan [21] and other researchers did not use total bilirubin as an independent predictor of CBC occurrence, because ALP and TBIL increased simultaneously when CBC occurred, but the predictive value of ALP was much higher than that of TBIL. Nakeeb et al. [5] pointed out that jaundice and elevated total bilirubin levels were not risk factors for CBC, which was consistent with current data. Atahan et al. [20] pointed out that the incidence of CBC in male patients was higher than in female patients, which may be due to sampling errors caused by fewer samples. The results of many researchers such as Langer [18] and Safarioleas [19] were consistent with current results. There was no difference between men and women in terms of CBC occurrence. Bircan [21] found that because of the larger cysts in patients with single cyst and more pericystic walls in patients with single cyst size, single lesion hydatid is more likely to develop CBC than multiple lesions. However, the results of this study are consistent with those of Kilic et al. [14] and Kayaalp et al. [8]; the number of hydatids could not be used as a predictor of CBC occurrence. In current data, the majority of patients are middle-aged, with elderly and adolescent patients being a minority, so to prove whether age is related to bile leakage, we plan to increase sample size in future research.

## Conclusions

In summary, in this study the risk of CBC of cystic echinococcosis was quantitatively assessed by cyst diameter, ALP, GGT and whether cysts were located in the hilum of the liver. The results showed that the higher the total score, the greater the risk of CBC. Doctors can intervene clinically as early as possible according to risk factors, such as ERCP before operation, ENBD or T tube during operation for biliary decompression, so as to reduce the occurrence of biliary leakage and formation of biliary fistula after operation. In current data, we used the nomogram model to predict the occurrence of CBC in patients with HCE. The C-index of nomogram was 0.791 (95% CI, 0.736–0.845). The C-index verified by bootstrap is 0.746, indicating high prediction accuracy. This model makes doctors more intuitive in the clinical diagnosis and treatment of cystic echinococcosis and facilitates communication between doctors and patients.

There are some limitations in this current study. This is a retrospective single-center study, meaning that for the patients with cystic echinococcosis in our hospital, the judgment of whether the patients have CBC depends mainly on the investigation of surgical records. Thus, there may be some information bias and patient selection bias. In addition, the model is only verified internally. Therefore, in future research, we plan to include multiple hospitals, expand the sample size of cystic echinococcosis patients, further optimize the selection of statistically and clinically significant predictors based on the current research results, and improve the nomogram to predict the occurrence risk of CBC of cystic echinococcosis.

As far as we know, the nomogram model is the first model that combines clinical and imaging indicators to predict the occurrence of CBC, which provides the basis for individualized consultation and clinical treatment.
